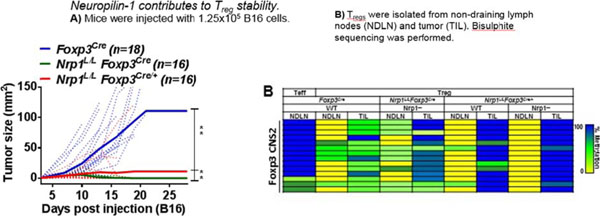# Elucidating the role of Neuropilin-1 in intra-tumoral regulatory T cell stability

**DOI:** 10.1186/2051-1426-3-S2-P274

**Published:** 2015-11-04

**Authors:** Abigail E Overacre, Maria Chikina, Greg M Delgoffe, Dario AA Vignali

**Affiliations:** 1 University of Pittsburgh, Pittsburgh, PA, USA

## 

Regulatory T cells (T_regs_) play an integral role in the adaptive immune system through suppression of self-reactive immune responses in order to prevent autoimmunity and maintain homeostasis. However, they are deleterious in cancer through suppression of the anti-tumor immune response. In fact, we show that deletion of 50% of T_regs_ results in normal tumor growth. Therefore, it is advantageous to understand the role of T_regs_ in the tumor microenvironment in order to create targeted cancer therapies. Our lab has shown that the Neuropilin-1 (Nrp1) pathway is required for T_reg_ stability in the tumor microenvironment, but is disposable for maintaining immune homeostasis in the periphery, identifying it as a prime therapeutic target.

In order to further understand the role of Nrp1-deficient T_regs_ intratumorally, we constructed a competitive environment by utilizing *Foxp3*, which is located on the X chromosome, and as a result of X-inactivation, female *Foxp3^Cre-YFP^* heterozygous mice are cellular heterozygotes. We generated *Nrp1^L/L^Foxp3^Cre-YFP/+^* heterozygous mice comprised of 50% WT T_regs_ and 50% Nrp1-deficient T_regs_. Surprisingly, when given B16 melanoma, heterozygous mice **phenocopy***Nrp1^L/L^Foxp3^Cre-YFP^* homozygous mice (Figure 1A). This suggests that **Nrp1-deficient T_regs_ are playing an active role in shifting the anti-tumor immune response by destabilizing surrounding WT T_regs_ as determined by DNA methylation status** (Figure 1B). Neither WT nor Nrp1-deficient T_regs_ in the tumor from *Nrp1^L/L^Foxp3^Cre-YFP/+^* mice can suppress in a standard microsuppression assay *ex vivo*, unlike WT T_regs_ from *Foxp3^Cre-YFP^* mice. Through various co-culture experiments, we revealed that destabilization of WT T_regs_ is possibly due to a soluble factor derived from Nrp1-deficient T_regs_. Our data revealed that Nrp1-deficient T_regs_ produce large amounts of IFNγ in the tumor microenvironment. Indeed, **when treated with IFNγ, WT T_regs_ lose suppressive capacity**. In order to uncover potential novel pathways leading to this phenotype, we are performing global transcript studies using RNASeq. Overall, we have shown that Nrp1 is required for intratumoral T_reg_ stability, and in its absence, there is an alteration in the tumor microenvironment, leading to an **enhanced anti-tumor immune response**. These studies uncover a novel potential target for future cancer immunotherapies that preserves peripheral immune health.

**Figure 1:**